# Self-Reported Sitting Time, Physical Activity and Fibrinolytic and Other Novel Cardio-Metabolic Biomarkers in Active Swedish Seniors

**DOI:** 10.1371/journal.pone.0163409

**Published:** 2016-09-22

**Authors:** Bethany J. Howard, Anita Hurtig-Wennlöf, Lovisa A. Olsson, Torbjörn K. Nilsson, David W. Dunstan, Patrik Wennberg

**Affiliations:** 1 Baker IDI Heart and Diabetes Institute, Melbourne, Australia; 2 School of Public Health & Preventive Medicine, Monash University, Melbourne, Australia; 3 Faculty of Medicine and Health, School of Health Sciences, Örebro University, Örebro, Sweden; 4 Department of Laboratory Medicine, Faculty of Medicine and Health, Örebro University, Örebro, Sweden; 5 Department of Medical Biosciences/Clinical Chemistry, Umeå University, Umeå, Sweden; 6 Mary MacKillop Institute for Health Research, Australian Catholic University, Melbourne, Australia; 7 School of Exercise and Nutrition Sciences, Deakin University, Melbourne, Australia; 8 School of Sports Science, Exercise and Health, The University of Western Australia, Perth, Australia; 9 School of Population Health, The University of Queensland, Brisbane, Australia; 10 Public Health and Clinical Medicine, Family Medicine, Umeå University, Umeå, Sweden; Universita degli Studi di Roma 'Foro Italico', ITALY

## Abstract

**Background:**

Too much sitting is linked with an increased risk of cardiovascular disease and mortality. The mediating mechanisms for these associations are largely unknown, however dysregulated fibrinolysis have emerged as a possible contributor.

**Objective:**

We examined the associations of self-reported overall sitting time and physical activity with fibrinolytic and other novel cardio-metabolic biomarkers in older adults.

**Materials and Methods:**

Data was analysed for 364 participants (74±7 yrs) of the Active Seniors group (retired, living independently in their own homes). Linear regression analyses examined associations of categories of categories of sitting time (≤3, 3–6, >6 hrs/day) and overall physical activity (Low, Moderate and High) with biomarkers in serum or plasma, adjusting for age, gender and smoking (with further adjustment for either overall physical activity or sitting time and BMI in secondary analyses).

**Results:**

Compared to sitting ≤ 3 hrs/day, sitting >6 hrs/day was associated with higher tissue plasminogen activator (tPA) and tissue plasminogen activator/plasminogen activator inhibitor-1 complex (tPA-PAI-1 complex). These associations were not independent of overall physical activity or BMI. Compared to those in the high physical activity, low physical activity was associated with a higher BMI, high-sensitivity C-reactive protein (hs-CRP) and tPA-PAI-1 complex levels. Only the associations of BMI and hs-CRP were independent of sitting time.

**Conclusions:**

These findings provide preliminary cross-sectional evidence for the relationships of sitting time with fibrinolytic markers in older adults. They also reinforce the importance of regular physical activity for cardio-metabolic health.

## Introduction

Sedentary behaviour, including prolonged sitting in the workplace, during commuting, and in the domestic environment, has emerged as a distinct cardiovascular risk factor, independent of leisure-time moderate- to vigorous-intensity physical activity (MVPA) levels [1]. Prospective studies have observed that sitting time is associated with an increased risk of cardiovascular morbidity and mortality [2]. Recent studies indicate that these detrimental associations may, in part, be mediated by mechanistic pathways beyond the traditional cardiovascular risk factors [3]. Specifically, evidence from cross-sectional studies showing adverse associations of various sedentary behaviours with the inflammatory marker C-reactive protein (CRP) and the inflammatory/haemostatic marker fibrinogen [4–7] support this hypothesis.

The inverse association of regular leisure-time physical activity with cardiovascular disease incidence and mortality is only partly mediated by its positive influence on traditional risk factors (e.g. stabilised weight, improved glucose metabolism, blood pressure and blood lipids) [8]. In a study of 3810 men in the UK, aged 69–74 years, self-reported physical activity showed a significant inverse association with blood levels of the fibrinolytic marker tissue plasminogen activator (tPA), as well as several other haemostatic markers [9]. The same men had been to an initial screening 20 years earlier and an examination of changes in physical activity over time showed that those who took up at least light physical activity had lower levels of tPA at the second examination compared to those who had remained inactive. Increased plasma levels of fibrinolytic markers, both inhibitors and activators of the fibrinolytic system, may reflect a thrombotic tendency and have been linked to coronary heart disease [10] and ischaemic stroke [11]. These findings suggest that alterations in fibrinolysis may be important additional mediating mechanisms for the benefits of physical activity on cardiovascular risk [12, 13].

As previous studies of the association between physical activity and fibrinolysis have focused on MVPA or the effect on fibrinolysis from more extreme situations of seated immobility [14], there is a lack of knowledge, especially in older adults, on how sedentary behaviour in daily life may impact on fibrinolytic activity. Gaining a greater understanding of the relationship between overall sitting time and fibrinolysis has important relevance for public health since objective measurement shows that older adults (aged ≥ 60 years) on average currently spends 65–80 percent of their waking time sitting [15] and are less likely than those of younger age to be regularly active [16]. The consequences from “too much sitting” on the fibrinolytic system may be of particular importance among elderly since an age-dependent decrease in fibrinolytic activity has been described [17].

The primary aim of this study was to examine the cross-sectional associations of sitting time and physical activity with other novel (fibrinolytic, inflammatory and apolipoproteins) and traditional cardio-metabolic biomarkers in a population of older adults living independently in their own home. This group represents: i) an important target for preventative efforts as the risk for cardiovascular events is increased in the age-group [18, 19] and ii) an optimal study population as they lack severe disabling diseases that may confound the association between sitting time and fibrinolysis.

## Materials and Methods

### Participants

Active Seniors (AS) were recruited by a sampling procedure aimed at an elderly retired population, living in various communities in central Sweden between May 2003 and August 2007 [20]. The locations for the recruitment were selected to represent a broad range of socioeconomic levels and included rural as well as urban and suburban areas. The sample consisted of 389 senior citizens recruited from several retired persons’ organisations. Being retired, living independently in their own homes in addition to participation in such organisations were the sole inclusion criteria, not preset health criteria. Participants are described as ‘Active Seniors’ in contrast to elderly persons that do not engage themselves in the mentioned social activity. All were Caucasians, most of them born in the 1920’s and 1930’s, mean age at sampling was 74 ± 5 years for both genders, and the gender ratio M/F was 127/262. Exclusion for missing data for exposures, covariates or biomarkers left 364 eligible participants. The study was approved by the Research Ethics Committee of the Örebro County Council and the Regional Ethical Review Board, Uppsala, Sweden, and was carried out in accordance with the Declaration of Helsinki. All participants provided written informed consent.

### Data Collection

#### Sitting Time and Physical Activity

Sitting Time and Physical Activity was assessed by a version of the International Physical Activity Questionnaire, the IPAQ-E [21], and self-administered. This modified version is based on the short version of the International Physical Activity Questionnaire (https://sites.google.com/site/theipaq/) developed and previously validated specifically for persons above 65 years [21]. Notable differences included the order of questions about the time spent in different levels of PA have been reversed and the provision of examples of activities that are more suited towards elders. Participants self-reported their sitting time in response to a single question concerning their time spent sitting per day in the last seven days. Categories of sitting time were derived based on tertile distributions as ≤3 hours per day, 3 to 6 hours per day and >6 hours per day. The categorical outcome from IPAQ-E assigns the subjects into three PA categories (Low, Moderate and High) based on the reported time, in combination with a weighting factor for the different activities (i.e. a factor 3.3 for walking, 4.0 for moderate PA and 8.0 for vigorous PA) (https://sites.google.com/site/theipaq/scoring-protocol).

#### Cardio-metabolic biomarkers

Venepuncture blood samples were taken, with the subjects in the supine position using vacuum tubes. Serum was obtained after clotting for 30 to 60 minutes at room temperature and centrifuging for 10 minutes at 2000g. All samples were stored at -80°C. The serum samples were analysed on a Hitachi 911 multianalyser, Roche, Mannheim, FRG. Apolipoprotein A-1 (APOA-1) and Apolipoprotein B (APO B) were analysed in serum using an immunoturbidimetry method and high sensitivity CRP (hs-CRP) was analysed using a latex enhanced immunoturbidimetry method CRP (Latex) HS, both from Roche/Boehringer Mannheim, FRG. LDL and HDL cholesterol were measured by direct homogeneous assays based on detergent treatment of the serum, N-geneous^™^ HDL-c and N-geneous^™^ LDL reagents, respectively, from Genzyme Corporation (Cambridge, MA, USA). Venous samples were drawn with a minimum of stasis in siliconized evacuated Stabilyte^™^ tubes (Biopool^®^, Umeå, Sweden) for plasma samples. The tPA, PAI-1 and tPA-PAI-1 complex concentrations in Stabilyte plasma [22] were determined using enzyme-linked immunosorbent assays using reagent kits from Biopool^®^ (Umeå, Sweden). All coefficients of variation were under 7.5%.

#### Anthropometrics

The participants were weighed wearing light indoor clothing but without shoes, and the weight was approximated to the nearest 0.1 kg. Body Mass Index (BMI) (kg/m^2^) was calculated using height and weight.

#### Blood pressure

Resting systolic and diastolic blood pressure were measured using a validated [23] automatic oscillometric method (Dinamap model XL Critikron, Inc., Tampa, Florida.). The subjects were in a sitting, relaxed position, and registrations were taken every minute for 4 minutes with the aim of obtaining a set of systolic registrations not varying more than 5 mmHg. A mean value of the last two registrations was used for both resting systolic and diastolic blood pressure, in mmHg.

### Statistical Analyses

Spearman rank correlation was used to investigate the correlation between sitting time and physical activity. Linear regression models examined associations of categorical daily sitting time and PA with continuously measured cardio-metabolic outcomes. We found no significant interaction between sitting time (or physical activity) and gender on levels of fibrinolytic markers. Model A was unadjusted and Model B was adjusted for age, gender and smoking. Where significant associations for Model B were observed, secondary analyses with further adjustment for either PA or sitting time and BMI were carried out (Model C and Model D). With the exception of BMI, diastolic blood pressure, LDL cholesterol, Apoliprotein B and Apolipoprotein A1, all outcomes were log-transformed to improve normality. Models did not display problems from non-normality or non-linearity. Analyses were conducted in STATA 12.1 (College Station, Texas, StataCorp). Significance was set at *P*<0.05.

## Results

Participant characteristics are described in Table 1. On average the participants were overweight (as indicated by a mean BMI of 26 **±** 3.8 kg/m^2^) with the majority spending 3–6 hrs sitting and reporting high physical activity which corresponds to 3 hours of vigorous physical activity or 12 hours of moderate physical activity per week.

**Table 1:** Characteristics of the study population.

	Mean (± SD) or % (n)
*n*	364
**Covariates**	
Age (yrs)	74 (6.8)
Current smoker (%)	6 (22)
**Behaviours**	
Sitting time ≤3hrs/day (%)	24 (88)
Sitting time 3-6hrs/day (%)	49 (178)
Sitting time >6hrs/day (%)	27 (98)
High physical activity (%)	53 (193)
Moderate physical activity (%)	32 (116)
Low physical activity (%)	15 (55)
**Cardio-metabolic biomarkers**	
BMI (kg/m^2^)	26 (3.8)
Systolic BP (mmHg)	148 (25)
Diastolic BP (mmHg)	77 (11)
HDL cholesterol (mmol/L)	1.64 (0.43)
LDL cholesterol (mmol/L)	3.52 (0.94)
ApoA-1 (g/L)	1.62 (0.3)
ApoB (g/L)	0.9 (0.2)
ApoB/ApoA-1 ratio (g/L)	0.58 (0.17)
hs-CRP (mg/L)	2.33 (2.81)
**Fibrinolytic biomarkers**	
tPA (μg/L)	12.2 (4.7)
PAI-1 (μg/L)	24.9 (16.9)
tPA-PAI-1 complex (μg/L)	8.1 (5.08)

### Sitting time

Sitting time was positively, albeit weakly, correlated with physical activity (r = 0.194, p<0.001). In the crude model (A), sitting time was significantly associated with BMI, systolic blood pressure, tPA and tPA-PAI-1 complex (Table 2). Following further adjustment for age, gender and smoking only the associations with tPA and tPA-PAI-1 complex remained significant. Sitting more than six hours per day was associated with 12% (95% CI: 1–24%) higher tPA and 19% (1–41%) higher tPA-PAI-1 complex, relative to those who sat less than or equal to three hours per day (Table 2). These associations were no longer significant after adjustment for physical activity and BMI.

**Table 2:** Associations of sitting time with cardio-metabolic and fibrinolytic biomarkers.

		Sitting time		
	Model	3–6 hrs/day	>6 hrs/day	P for trend
**BMI (kg/m**^**2**^**)**	**A**	0.97 (-0.01, 1.95)	1.13 (0.03, 2.23)*	0.048
	**B**	0.95 (-0.04, 1.94)	1.12 (-0.01, 2.25)	0.056
**Systolic BP (mmHg)**†	**A**	1.01 (0.97, 1.06)	1.06 (1.01, 1.11)*	0.020
	**B**	1.01 (0.97, 1.05)	1.04 (0.99, 1.09)	0.085
**Diastolic BP (mmHg)**	**A**	0.73 (-2.1, 3.55)	0.76 (-2.43, 3.94)	0.648
	**B**	0.15 (-2.68, 2.97)	0.13 (-3.09, 3.35)	0.938
**LDL cholesterol (mmHg)**	**A**	-0.15 (-0.39, 0.09)	-0.15 (-0.42, 0.12)	0.286
	**B**	-0.12 (-0.37, 0.12)	-0.12 (-0.4, 0.16)	0.408
**HDL cholesterol (mmHg)**†	**A**	0.95 (0.88, 1.01)	0.94 (0.87, 1.01)	0.118
	**B**	0.97 (0.91, 1.03)	0.95 (0.88, 1.01)	0.120
**ApoA-1 (g/L)**	**A**	-0.05 (-0.12, 0.03)	-0.03 (-0.11, 0.06)	0.571
	**B**	-0.02 (-0.09, 0.05)	-0.02 (-0.1, 0.06)	0.676
**ApoB-1 (g/L)**	**A**	-0.03 (-0.09, 0.02)	-0.03 (-0.09, 0.03)	0.340
	**B**	-0.03 (-0.08, 0.02)	-0.03 (-0.09, 0.03)	0.374
**ApoB/ApoA-1 ratio (g/L)**	**A**	-0.02 (-0.06, 0.03)	-0.03 (-0.08, 0.02)	0.264
	**B**	-0.02 (-0.07, 0.02)	-0.03 (-0.08, 0.02)	0.255
**hs-CRP (mg/L)** †	**A**	1.04 (0.82, 1.32)	0.95 (0.73, 1.24)	0.680
	**B**	0.99 (0.78, 1.26)	0.88 (0.67, 1.15)	0.337
**Fibrinolytic biomarkers**				
**tPA (μg/L)** †	**A**	1.07 (0.97, 1.18)	1.19 (1.07, 1.32)**	0.002
	**B**	1.03 (0.94, 1.13)	1.12 (1.01, 1.24)*	0.036
	**C**	1.03 (0.94, 1,13)	1.10 (0.99, 1.23)	0.067
	**D**	0.99 (0.91, 1.08)	1.06 (0.97, 1.17)	0.205
**PAI-1 (μg/L)** †	**A**	1.04 (0.92, 1.19)	1.07 (0.92, 1.24)	0.386
	**B**	1.03 (0.9, 1.17)	1.03 (0.89, 1.2)	0.659
**tPA-PAI-1 complex (μg/L)** †	**A**	1.11 (0.95, 1.29)	1.28 (1.07, 1.52)**	0.005
	**B**	1.06 (0.92, 1.24)	1.19 (1, 1.41)*	0.048
	**C**	1.06 (0.91, 1.23)	1.16 (0.97, 1.38)	0.103
	**D**	1.00 (0.87, 1.14)	1.09 (0.93, 1.27)	0.289

Data are β Coefficient (95% CI), the absolute difference of the biomarker level compared to the reference category: ≤3hrs sitting time per day, or

^†^ Relative Rate (back-transformed from the log scale), the relative difference of the biomarker level compared to the reference category: ≤3hrs sitting time per day. Model A: unadjusted, Model B: adjusted for age, gender and smoking, Model C: adjusted for Model B and for physical activity, Model D: adjusted for Model C and for BMI.

**Statistical significance p<0.01

*Statistical significance p<0.05.

### Physical activity

In the crude model (A), physical activity was significantly associated with BMI, systolic blood pressure, hs-CRP, tPA, tPA-PAI-1 complex (Table 3). Following further adjustment for age, gender and smoking, only the associations with BMI, hs-CRP and tPA-PAI-1 complex remained significant (Table 3). Compared to those who were highly physically active, low physical activity was associated with a 1.6 kg/m^2^ (0.5–2.8) higher BMI and a 21% (1–45) higher plasma level of tPA-PAI-1 complex (Table 3). The association between physical activity and BMI remained significant after further adjustment for sitting time (β 1.5 [95% CI: 0.3–2.7]), but the association with tPA-PAI-1 complex did not. Independent from sitting time, reporting moderate or low physical activity was associated with a 29% (4–59, p = 0.021) and 42% (7–89, p = 0.015) higher respective level of hs-CRP, compared to those reporting high physical activity. Only the association between moderate physical activity and hs-CRP was independent from BMI (moderate PA: 29% [5–58], p = 0.016; low PA: 29% [-2–69], p = 0.071).

**Table 3:** Associations of physical activity with cardio-metabolic and fibrinolytic biomarkers.

		Physical Activity (IPAQ category)		
	Model	Moderate	Low	P for trend
**BMI (kg/m**^**2**^**)**	**A**	0.08 (-0.79, 0.96)	1.64 (0.49, 2.78)*	0.017
	**B**	0.07 (-0.81, 0.95)	1.63 (0.46, 2.80)**	0.021
	**C**	-0.01 (-0.89, 0.88)	1.48 (0.30, 2.66)*	0.045
**Systolic BP (mmHg)**†	**A**	1.03 (0.99, 1.07)	1.05 (1.00, 1.11)*	0.024
	**B**	1.03 (0.99, 1.07)	1.02 (0.97, 1.07)	0.195
**Diastolic BP (mmHg)**	**A**	1.38 (-1.16, 3.92)	2.26 (-1.05, 5.56)	0.132
	**B**	1.23 (-1.29, 3.75)	1.67 (-1.66, 5.01)	0.239
**LDL cholesterol (mmHg)**	**A**	0 (-0.21, 0.22)	-0.28 (-0.56, 0)	0.103
	**B**	0.01 (-0.21, 0.23)	-0.26 (-0.54, 0.03)	0.157
**HDL cholesterol (mmHg)**†	**A**	0.97 (0.92, 1.03)	0.93 (0.86, 1.01)	0.077
	**B**	0.98 (0.93, 1.04)	0.93 (0.86, 1.00)*	0.049
	**C**	0.98 (0.93, 1.04)	0.93 (0.87, 1.01)	0.085
	**D**	0.98 (0.93, 1.04)	0.96 (0.89, 1.03)	0.228
**ApoA-1 (g/L)**	**A**	0.01 (-0.06, 0.08)	-0.04 (-0.13, 0.05)	0.471
	**B**	0.01 (-0.05, 0.08)	-0.05 (-0.13, 0.03)	0.416
**ApoB-1 (g/L)**	**A**	0.02 (-0.03, 0.06)	-0.02 (-0.08, 0.04)	0.713
	**B**	0.02 (-0.03, 0.06)	-0.02 (-0.08, 0.04)	0.870
**ApoB/ApoA-1 ratio (g/L)**	**A**	0.01 (-0.04, 0.05)	0.01 (-0.05, 0.06)	0.793
	**B**	0 (-0.03, 0.04)	0.01 (-0.04, 0.07)	0.608
**hs-CRP (mg/L)** †	**A**	1.26 (1.02, 1.56)*	1.43 (1.09, 1.89)*	0.004
	**B**	1.26 (1.02, 1.56)*	1.38 (1.04, 1.82)*	0.008
	**C**	1.29 (1.04, 1.59)*	1.42 (1.07, 1.89)*	0.004
	**D**	1.29 (1.05, 1.58)*	1.29 (0.98, 1.69)	0.017
**Fibrinolytic biomarkers**				
**tPA (μg/L)** †	**A**	1.02 (0.93, 1.11)	1.20 (1.07, 1.34)**	0.006
	**B**	1.01 (0.93, 1.09)	1.12 (1.00, 1.25)*	0.087
	**C**	1.00 (0.92, 1.08)	1.10 (0.99, 1.23)	0.172
	**D**	1.00 (0.93, 1.07)	1.03 (0.94, 1.14)	0.609
**PAI-1 (μg/L)** †	**A**	1.04 (0.92, 1.17)	1.06 (0.91, 1.23)	0.420
	**B**	1.03 (0.91, 1.15)	1.00 (0.86, 1.17)	0.833
**tPA-PAI-1 complex (μg/L)** †	**A**	1.08 (0.94, 1.25)	1.31 (1.09, 1.57)**	0.004
	**B**	1.08 (0.94, 1.23)	1.21 (1.01, 1.45)*	0.032
	**C**	1.06 (0.93, 1.22)	1.18 (0.99, 1.42)	0.067
	**D**	1.06 (0.94, 1.20)	1.07 (0.91, 1.26)	0.294

Data are β Coefficient (95% CI) the absolute difference of the biomarker level compared to the reference category: high physical activity or

^†^Relative Rate (back-transformed from the log scale), the relative difference of the biomarker level compared to the reference category: high physical activity. Model A: unadjusted, Model B: adjusted for age, gender and smoking, Model C: adjusted for Model B and for sitting time, Model D: adjusted for Model C and for BMI.

**Statistical significance p<0.01

*Statistical significance p<0.05.

## Discussion

The main findings of this study were that sitting more than six hours per day was associated with higher plasma levels of tPA and tPA-PAI-1 complex but not with hs-CRP, relative to those who sat less than three hours per day. The associations with tPA and tPA-PAI-1 complex were independent of age, gender and smoking, but not independent of physical activity or BMI. Physical activity was inversely associated with BMI and plasma levels of hs-CRP and tPA-PAI-1 complex, independently of age, gender and smoking.

To our knowledge, this is the first study to examine the relationship between overall sitting time and blood levels of tPA, PAI-1 and tPA-PAI-1 complex, key markers of the fibrinolytic system, in an older adult sample. However, associations of sitting time with plasma levels of fibrinolytic markers tPA and tPA-PAI-1 complex were attenuated to the null with further adjustment for physical activity and BMI. There are likely to be a number of reasons for this. Firstly, obesity is considered to be a part of a potentially causal pathway related to the adverse associations of sedentary behaviour [24, 25] and has been linked to dysregulated fibrinolysis [26]. Obesity could therefore be considered as a potential mediator rather than a confounder [27]. Secondly, the physical activity measure in the current study included physical activity regardless of intensity (both LPA and MVPA). Previous research has shown strong inverse correlations between LPA and sitting time [28] (more time spent in LPA is associated with less time spent sedentary).

In the current study there was no association of sitting time with CRP. In contrast, previous studies have reported associations of various sedentary behaviours with CRP [4–7]. In an analysis of the 1958 British birth cohort, Pinto Pereira et al. [4] found that higher television-viewing time was associated with higher levels of CRP and fibrinogen in 45 year old women, whereas work sitting was not. In a sample of adults with a mean age 59 years, Yates et al. [5] found that overall sitting time was associated with CRP levels in women but not in men. Gennuso et al. [6] used activity monitors to objectively assess sedentary time in an older population of men and women, mean aged 75 years and more comparable to the current study. They found positive associations between sedentary time and levels of CRP, independent of the level of MVPA. A possible explanation for the discrepancy between the current study and previous findings may be the differences in sedentary behaviour measures (TV viewing and workplace sitting) [4] and/or the comparatively low levels of sitting time and high levels of physical activity in our sample. Furthermore, the small sample size may have limited the ability to detect an association.

Inflammation is considered to play a key role in the atherosclerosis process and CRP is currently the most validated inflammatory biomarker [29]. A meta-analysis reported that CRP concentrations have continuous associations with coronary heart disease, ischaemic stroke and vascular as well as non-vascular mortality [30]. The associations of physical activity with CRP and tPA-PAI-1 complex in this study supports previous research suggesting that the beneficial effect of regular physical activity on cardiovascular incidence, to a substantial part, may be mediated by mechanistic pathways beyond the traditional cardiovascular risk factors [9, 12, 13, 31]. In a study of over 27.000 women, Mora et al. [12] found that the difference between levels of physical activity in inflammatory and haemostatic markers could explain about one third of the inverse relationship between physical activity and cardiovascular disease [12].

The relationship between sitting time (or other measures of sedentary behaviour) and MVPA are in general, weak or non-existent and may differ between age-groups and gender. For example, in a population-based sample of older men and women from the AusDiab study, there was a significant but weak negative correlation between sitting time and MVPA in women, but not in men [32]. In contrast, sitting time was weakly but positively correlated with physical activity in our sample. This discrepancy may be explained by the sampling procedure. Our sample consisted of ‘Active Seniors’ with a high proportion of physically active participants and comparatively low levels of sitting time. A population-based sample would probably have resulted in a higher proportion of individuals with both low levels of physical activity and high levels of sitting time.

There are several limitations with the study. Causal inferences are not possible as this is a cross-sectional study and our findings need to be further investigated in prospective and interventional trials. Sitting time and physical activity were self-reported which may increase the risk for misclassification bias. As other subjective measurements, the questionnaire may have a limited validity for measuring intensities, especially light intensity physical activity. Also, previous research has suggested that self-reported total sitting time may underestimate accelerometer-derived sitting time in older adults [33]. However, the questionnaire used in this study has been developed specifically for persons over 65 years and shown to be valid at a population level [21]. Another limitation is the sample size which restricted the statistical power of the study. The potential for residual confounding from unmeasured factors such as diet, alcohol, pharmacological treatments and socio-economic position can also not be excluded.

## Conclusions

In a sample of older adults, preliminary cross-sectional evidence was found for the relationships of sitting time with plasma levels of fibrinolytic markers tPA and tPA-PAI-1 complex. Our findings add to the previous research on the proinflammatory aspects of “too much sitting” [2] by suggesting that dysregulated fibrinolysis may be an additional mechanism that contributes to the deleterious associations of sitting time with cardiovascular disease. Low physical activity was associated with higher BMI and higher plasma levels of hs-CRP and tPA-PAI-1 complex. The findings in this study provide further evidence to the potential importance of reducing sitting time and participating in regular physical activity to improve cardio-metabolic health.
